# Mechanical Design, Control, and Laboratory Test of a Two-Degrees-of-Freedom Elbow Prosthesis

**DOI:** 10.3390/bioengineering12070695

**Published:** 2025-06-25

**Authors:** Ramsés Hernández-Cerero, Juan Alejandro Flores-Campos, José Juan Mojica-Martínez, Adolfo Angel Casarez-Duran, Luis Angel Guerrero-Hernández, Christopher René Torres-SanMiguel

**Affiliations:** 1 Instituto Politécnico Nacional, Escuela Superior de Ingeniería Mecánica y Eléctrica, Sección de Estudios de Posgrado e Investigación, Unidad Zacatenco, Ciudad de México 07738, Mexico; 2 Instituto Politécnico Nacional, Unidad Profesional Interdisciplinaria en Ingeniería y Tecnologías Avanzadas, Ciudad de México 07340, Mexico

**Keywords:** biomechanics, 2 degrees of freedom, elbow prosthesis, SMC-TBG control method

## Abstract

This study presents the design and experimental testing of a two-degrees-of-freedom (2DOF) elbow prosthesis prototype designed to replicate the movement patterns of a native or normal human elbow. Two methods of the control of the prosthesis, namely, the proportional–integral–derivative method (PID; a well-established method) and a combination of sliding mode control with a time base generator strategy (SMC + TBG; an advanced method), were compared on the basis of various performance metrics of the prosthesis, as obtained in laboratory tests. Among these metrics were the angular displacement and velocity as a function of time. The mechanical design combined 3D-printed components with custom-designed joints, featuring a worm gear transmission with a crown gear for flexion–extension, enhanced by torsional springs, and a pinion gear with a crown gear for pronation–supination and control. Sensors for voltage and current data acquisition enabled real-time monitoring and control. The prosthesis was tested in the laboratory with a range of motion of 100–120° for flexion–extension, 50° for supination, and 75° for pronation, demonstrating the adaptability of the actuators and validating their autonomy through battery-powered operation. The results showed that control using SMC + TBG resulted in biomimetic patterns for angular displacement and angular velocity of the prosthesis, whereas control using PID did not. Thus, the prosthesis with control provided using an SMC + TBG strategy may have been promised for use by people who have undergone transhumeral amputation.

## 1. Introduction

Most transhumeral amputees experience reduced prosthetic functionality due to control limitations, often relying on unconventional muscle groups, which leads to discomfort and inefficiency [1]. Elbow prostheses for this population typically offer two degrees of freedom (2DOF): flexion–extension and pronation–supination. These movements are essential for performing activities of daily living. Traditional mechanical designs—such as worm gears and crown gear mechanisms—enhance energy efficiency and minimize noise and wear through continuous tooth engagement [2,3]. While these solutions improve comfort and reduce vibration, the prosthetic experience is still limited by control strategies that lack adaptability and precision.

Current control methods include body-powered, myoelectric, and pneumatic systems, each with specific trade-offs in terms of usability, responsiveness, and power requirements [4]. PID (proportional–integral–derivative) control remains widely used due to its simplicity and ease of implementation [5]. However, it struggles with nonlinear system dynamics and often fails to generate smooth, biomimetic trajectories, resulting in abrupt or unnatural transitions [6,7,8]. An alternative approach is sliding mode control (SMC) combined with time base generation (TBG), which enhances robustness, reduces jerk, and generates bell-shaped velocity profiles [9,10]. This control strategy ensures smoother movements and adapts more effectively to system uncertainties, thereby improving rehabilitation outcomes [11,12,13]. Despite these advantages, few studies have directly compared SMC + TBG with traditional PID controllers in the context of elbow prostheses with two degrees of freedom (DOF).

**Figure 1:**
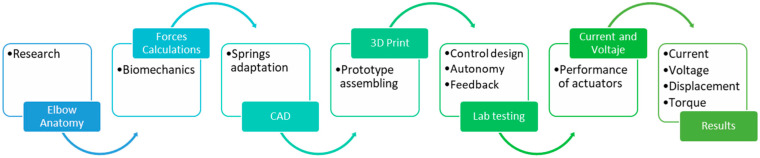
A flowchart of the steps in the development, fabrication, and experimental testing of the prosthesis.

This study aims to fill that gap by presenting the design, control implementation, and laboratory testing of a novel 2DOF elbow prosthesis (Figure 1). The prototype integrates mechanical, electronic, and control subsystems using 3D-printed parts, geared transmissions, and torsional springs [14,15,16]. An Arduino Nano^®^ was used to implement both control strategies—PID and SMC + TBG—alongside PWM (pulse width modulation) signal generation and Bluetooth-based remote control [17,18]. The performance was evaluated based on key metrics, including angular displacement over time, angular velocity, current consumption, and voltage regulation [19]. The results show that the SMC + TBG strategy yields more stable and natural joint behavior, reinforcing its potential for future use in rehabilitation and clinical settings.

## 2. Materials and Methods

This study evaluates a two-degrees-of-freedom elbow prosthesis, measuring current, displacement, and velocity during flexion–extension (0° to 120°) and pronation–supination, revealing smooth, coordinated patterns within healthy ranges. Current modulation, regulated by a PID controller, ensures stability, while SMC + TBG enhances robustness and generates biomimetic trajectories for natural, precise movements. This integration enhances adaptability, making it particularly suitable for transhumeral amputees. SMC + TBG complements the PID controller, optimizing current modulation to minimize mechanical wear and enhance energy efficiency. Current graphs reflect controlled power consumption, with peaks indicating high activity and valleys representing lower energy demands, validating system efficiency and synchronization. The integration of SMC + TBG with PID control significantly improves prosthesis performance, ensuring precise, natural, and stable movements. This approach enhances energy efficiency and mechanical durability, demonstrating its potential for advanced prosthetic and robotic systems.

### 2.1. Prosthesis Design

**Figure 2:**
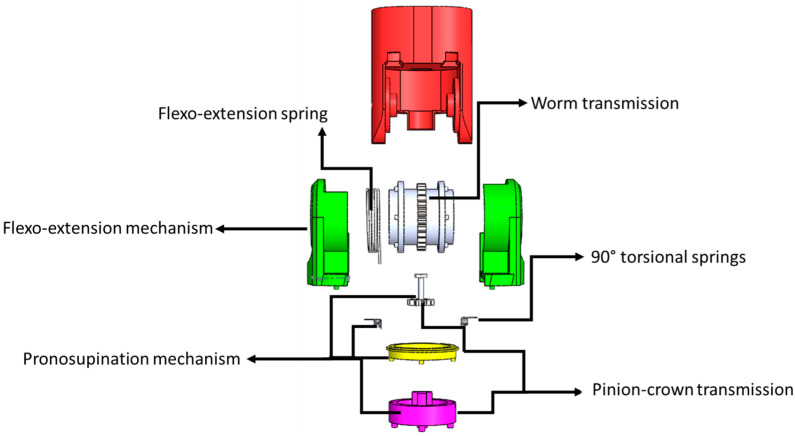
Schematic drawing of the prosthesis.

The innovativeness of the prosthesis lies in two aspects, namely, mechanical design (Figure 2) and control method. The flexion–extension mechanism uses a hinge-type apparatus with a worm gear and torsion spring, while the pronation–supination mechanism employs a pinion–crown gear and two torsional springs to enhance load capacity, store energy, and reduce motor torque. The control strategy employs a combination of sliding mode control with time base generation (SMC + TBG), ensuring precise, robust, and biomimetic movements. This combination optimizes performance, making the prototype ideal for applications requiring natural and adaptive motion [20]. The mechanical design of the prototype involved calculating components for the transmission system. For the worm and crown mechanism, the module, the number of starts, and crown gear teeth were determined to ensure proper sizing and mechanical performance. The pronation–supination movement utilizes a planetary transmission designed with data on gear teeth, module, pitch diameter, and transmission ratio (1.7, rounded to 2), which functions as a speed multiplier. This configuration adheres to the typical range of motion of the human upper limb, ensuring precise angular movements [21].

### 2.2. Mechanical Design

This study enables two degrees of freedom: flexion–extension (0–100°) and pronation–supination (0–50° supination, 0–75° pronation). Prototype design involved analyzing existing models’ actuator performance and integrating flexible actuators, such as springs, to enhance the replication of natural motion [22]. This approach ensures functionality within specified ranges and improves adaptability for rehabilitation applications.

#### Drives for Movements

A hinge-type mechanism was employed to replicate the flexion–extension movement. Given the requirements of this motion, a worm and crown gear transmission was selected due to its ability to effectively facilitate the desired movement [23]. This configuration operates with minimal noise and emulates the kinematic properties of the elbow joint. The drive speed ratio was carefully designed to range between 40 and 60 RPM, aligning with the natural speeds and timing of the elbow joint. A high gear ratio was prioritized to ensure the transmission could fit within a compact space while supporting substantial loads. This design choice is further justified by the high friction generated by the worm and crown gear, which eliminates backlash and makes it ideal for the prototype’s requirements, such as load-bearing capabilities [24].

### 2.3. Kinematics

**Figure 3:**
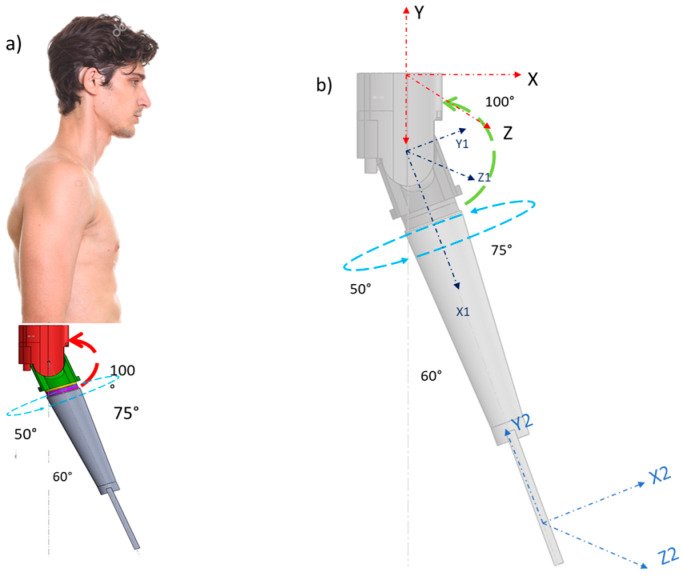
Boundary conditions: (**a**) prosthesis kinematics; (**b**) movement of the prosthesis.

To optimize the transmission design, forces in the flexion–extension movement were analyzed, focusing on the worm gear and worm wheel. The transmission ratio, defined as output speed divided by input speed, determines if the system is reductional (<1) or multiplier (>1) [22]. The motor operates at 440 RPM, reduced to 16 RPM via the worm and crown gear, engaging one tooth per worm rotation [25]. This ensures precise control over flexion–extension, meeting the prototype’s functional requirements. To effectively model the proposed elbow prosthesis prototype, the traditional two-axis plane was used as a reference. The control solution adopted is based on a pendulum with a torsional spring. The kinematic equation was derived from the potential energy equation, and Lagrange’s method was employed to describe the system’s motion. The initial condition was set at 300° (5/3π rad), and the final position was defined at 45° (¼π rad). The proposed model is illustrated in Figure 3b [26]. This approach ensures a robust representation of the system’s dynamics, facilitating accurate control and simulation of the elbow prosthesis.

Obtention of the transfer function and the error.

(1) $$\tau_{2}+k\Lambda q+\frac{N_{1}}{N_{2}}=\frac{d}{dt}\left(\frac{\partial L}{\partial \overset{•}{q}}\right)-\frac{\partial L}{\partial q_{2}}$$

(2) $$\tau_{C}=-Kpe-Kd\dot{e}+G\left(q\right)$$

(3) e=q−qd

Figure 3 compares a conventional single-axis elbow prosthesis with a 2-DOF prototype, which incorporates pronation–supination to provide a more natural range of motion. The Denavit–Hartenberg (D-H) equations model the 2-DOF mechanism: θ_1_ for flexion–extension (humerus, *L*_1_) and θ_2_ for pronation–supination (forearm, *L*_2_). Table 1 summarizes performance parameters, ensuring accurate kinematics for a functional and adaptive prosthesis.

A two-degrees-of-freedom (2-DOF) elbow prosthesis allows for two main movements: flexion–extension (FE), which is a sagittal plane movement similar to that of a hinge, and pronation–supination (PS), which is the rotation of the forearm around its longitudinal axis. In this case, the prosthesis does not use the humerus as a reference, meaning that the base coordinate system is not fixed to this bone. Additionally, pronation–supination does not occur at the wrist, suggesting that the axis of rotation is located within a structure inside the prosthesis, possibly near the ulna and the radius. To describe the motion of this prosthesis, the Denavit–Hartenberg (DH) notation was used, which allows for modeling the links and joints of a robotic manipulator. Since the prosthesis has two degrees of freedom, it is modeled with two rotational joints (R-R). The final effector defined by

(4) $$T_{2}^{0}=T_{1}^{0}\cdot \;T_{2}^{1}$$

The following operation determines the position of the final effector.

(5) $$T_{2}^{0}=\left[\begin{array}{cccc} \cos\theta_{1}\cos\theta_{2} & -\cos\theta_{1}\sin\theta_{2} & \sin\theta_{1}\; & \alpha_{i}\cos\theta_{1}+\alpha_{ii}\cos\theta_{1}\cos\theta_{2}\\ \sin\theta_{1}\cos\theta_{2} & -\sin\theta_{1}\sin\theta_{1} & -\cos\theta_{1}\; & \alpha_{i}\sin\theta_{1}+\alpha_{ii}\sin\theta_{1}\cos\theta_{2}\\ \sin\theta_{2} & \cos\theta_{2} & 0 & \alpha_{2}\sin\theta_{2}\\ 0 & 0 & 0 & 1 \end{array}\right]$$

The orientation of the elbow prosthesis is critical for understanding its spatial configuration and operational capabilities. In the Denavit–Hartenberg representation, the orientation is encoded within the final effector position.

(6) $$x2=\alpha_{i}\cos\theta_{1}+\alpha_{ii}\cos\theta_{1}\cos\theta_{2}$$

(7) $$y2=\alpha_{i}\sin\theta_{1}+\alpha_{ii}\sin\theta_{1}\cos\theta_{2}$$

(8) $$z2=\alpha_{ii}\sin\theta_{2}$$

Equation (8) describes the final transformation matrix for the flexo-extension movement trajectory.

### 2.4. Mechanical Parameters Determined

**Figure 4:**
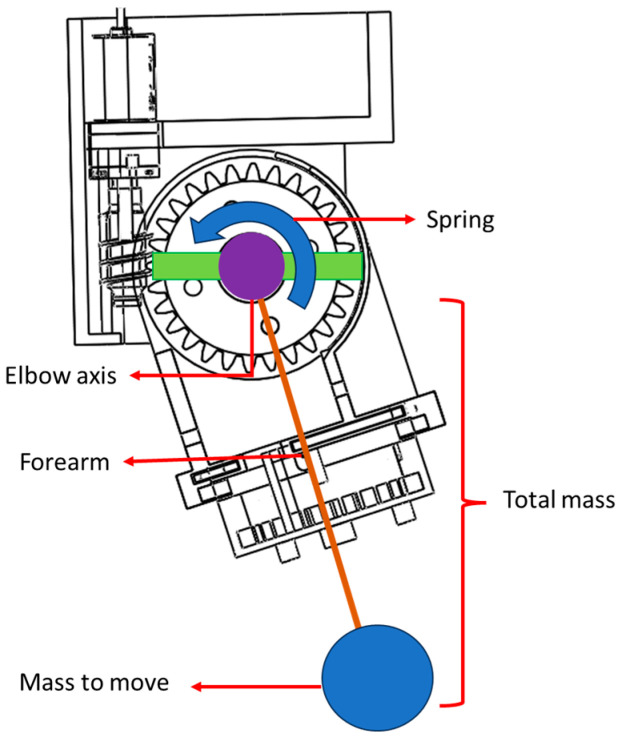
Transmission scheme.

In this work, key parameters for the experimental setup include the total mass (comprising the loaded mass and forearm load), forearm length, spring constant, transmission ratio of gearbox, gravity, and moment of inertia. Figure 4 illustrates the elements considered during the simulation and laboratory testing of the prototype, ensuring accurate replication of the flexion–extension movement and validating the system’s functionality under controlled conditions.

### 2.5. Control Strategies

**Figure 5:**
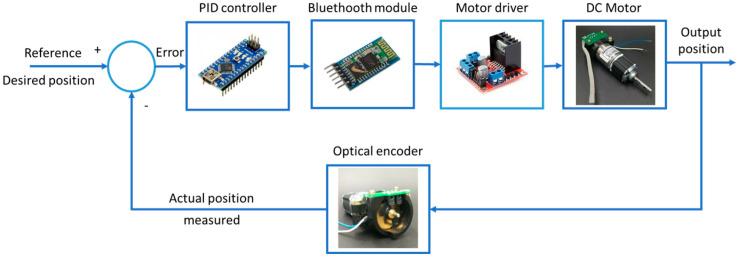
A block diagram for the flexion–extension position.

The SMC component provides robustness against uncertainties and disturbances, while TBG generates smooth velocity profiles, enabling controlled and fluid transitions in movements. This combination optimizes rehabilitation, reduces muscle fatigue, and enhances adaptability in applications requiring precise motion control. A key challenge in implementing the SMC-TBG control strategy in elbow prostheses is accurately modeling bone rotation and its impact on forearm mobility, particularly during pronation–supination movements between the radius and ulna. This aspect, which has not been addressed in previous studies such as those of [27,28], is essential for replicating coordinated and natural movements. The combination of TBG with SMC would not only synchronize the hand’s trajectory but also precisely model the dynamics of pronation–supination, enhancing the prosthesis’s functionality. This approach represents a significant improvement over traditional control methods, offering a more comprehensive and adaptive solution for rehabilitation applications. The objective of this experiment is to evaluate the accuracy of angular position control for a DC-geared motor using a PID controller and the SMC + TBG control method. An Arduino board controls a 12 V brushed Namiki DC motor, while the LN298N dual H-bridge driver generates the signal and manages direction control. For the pronation–supination movement, a stepper motor with its respective driver is utilized, enabling motion within the functional range designed for the prototype. The implementation includes PID tuning algorithms to generate precise signal outputs for both motions. This setup ensures accurate control of the DC motor and stepper positions, which is crucial for precision control systems. The function of a motor position controller is to interpret a signal representing the desired angle and to direct the motor to that position. Figure 5 illustrates the controlled conditions.

The control strategy applies in the articular space by means of the pseudoinverse of the Jacobian:

(9) τ=−KJ+(q)sx

### 2.6. Autonomy of the Prototype

**Figure 6:**
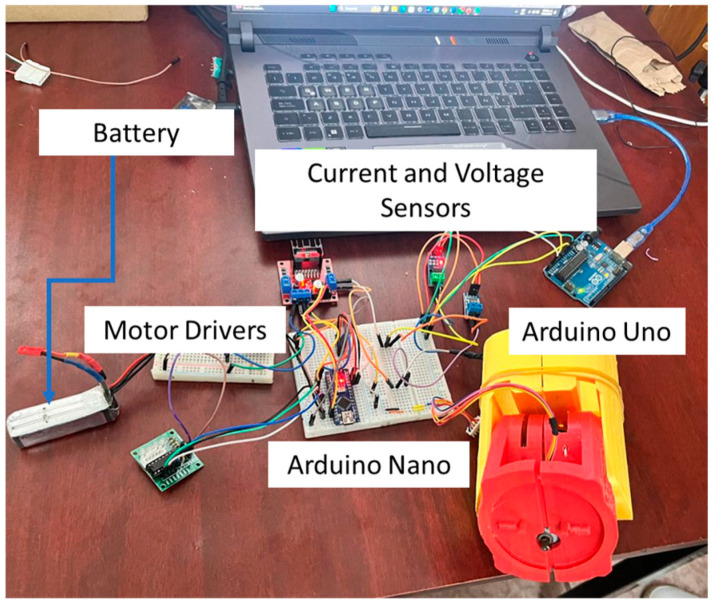
Experimental setup for autonomy testing for a control system.

To ensure the autonomy and practicality of the prosthesis, a rechargeable battery-based power supply was used, as shown in Figure 6. A Tattu 3-cell battery was selected, delivering 11.1 V per cell, with a total output of 5 Wh and a capacity of 450 mAh. This setup balances high energy density and low weight, thereby allowing precise and responsive movements while maintaining user comfort. The battery supports prolonged usage, reducing the frequency of recharging and enhancing the user experience. Its integration was carefully planned for efficient control and power management, ensuring seamless functionality and reliability in real-world scenarios. This solution significantly contributes to the prototype’s autonomy and usability.

## 3. Results and Discussion

**Figure 7:**
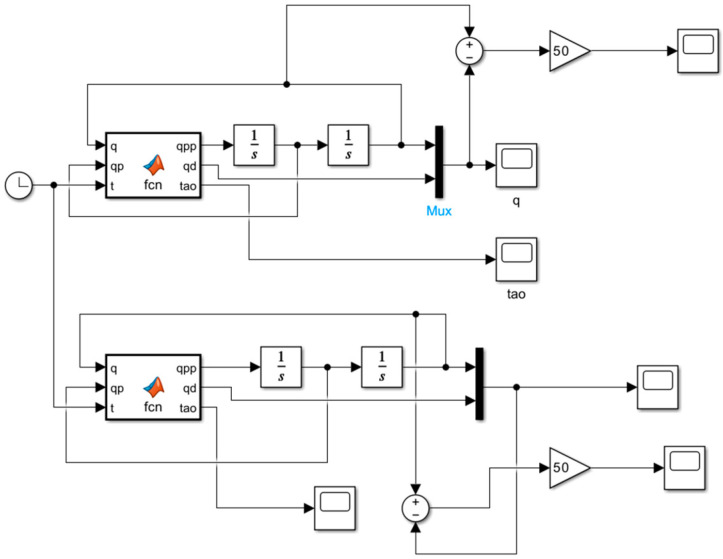
Simulink model of a 2-DOF elbow joint controlled with SMC + TBG.

The experiments were conducted using Simulink R2024b^®^ software and served to illustrate the behavior of the prototype, taking into account the torque generated by the motors, the effects of air resistance, the influence of gravity, and the weight of the load to be supported, and allow replication of any movement (Figure 7). In addition to the previously mentioned tests, an experiment was conducted on the prototype, considering the operating cycles over a specific period. The movements must be completed within a two-second time frame, with the actuators receiving sinusoidal signals reflecting the pulses for each motion. The block diagram represents a mass-torsional spring system in an open-loop configuration, allowing replication of any movement, as shown in Figure 7. The transfer function, derived from the mathematical model in the hardware design, supports this system. This section provides a concise description of the experimental results, their interpretation, and the conclusions drawn from them.

**Figure 8:**
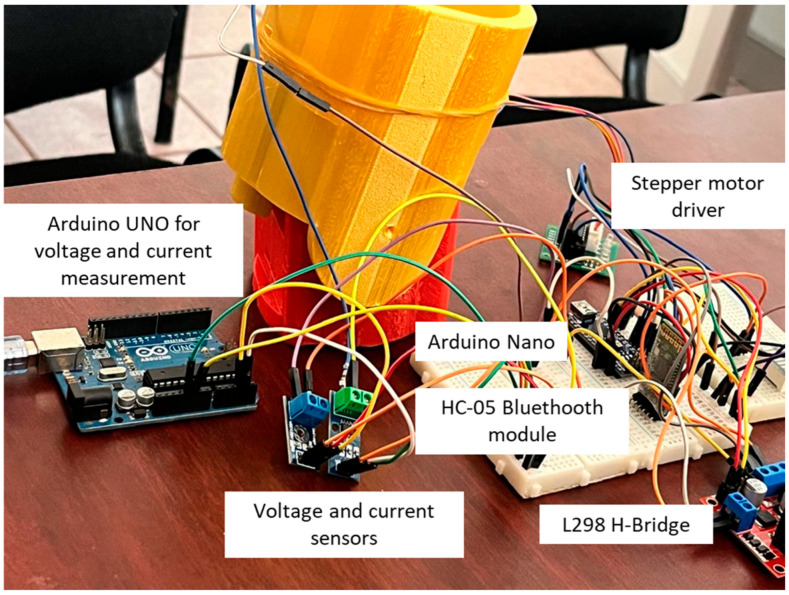
Electronic setup for Arduino.

The simulation experiments yielded position and velocity signals. Figure 8 illustrates the first stage of connecting the circuit, marking the commencement of the manufacturing and assembly process, which encompasses the design, construction, and assembly stages necessary for functionality. The integration of sliding mode control with time base generation (SMC + TBG) enhances the process by ensuring accurate tracking and optimization of signals for smooth, natural movements.

SMC provides robustness against uncertainties, while TBG generates biomimetic trajectories with bell-shaped velocity profiles, ensuring precise and stable motion. During assembly, SMC + TBG ensures motors, springs, and mechanisms work harmoniously, maintaining precise control and adaptability under external loads. This demonstration showcases the adaptive capabilities and precise angle control of the elbow prototype under a 1 kg load, thereby establishing the relationship between the motor, mechanisms, and the spring, on the one hand, and the output angles and velocities, on the other hand. The SMC + TBG strategy is enhanced by SMC, which ensures stability and accuracy under external forces, and TBG, which generates smooth, biomimetic trajectories for natural movements. This combination maintains the desired angles and velocities under load changes. SMC + TBG optimizes the system, dynamically adjusting motor response to maintain desired angles and velocities under load. The prototype, manufactured using 3D printing with PLA (polylactic acid), was assembled and tested on a bench, validating its functionality and performance. This highlights its potential for advanced prosthetic and robotic applications requiring precise, natural motion.

**Figure 9:**
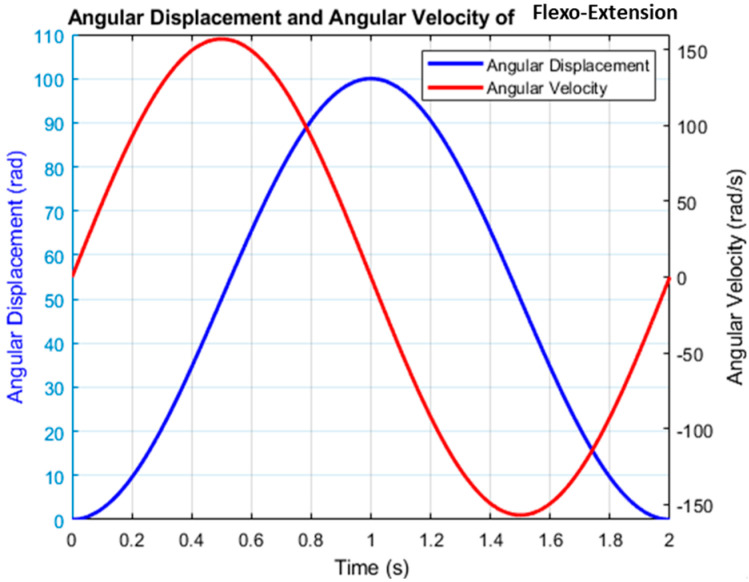
The flexo-extension displacement of the prototype testing.

The angular displacement (blue curve) represents the *θ*_1_ angle of flexion–extension (Figure 9), following the desired trajectory imposed by the SMC + TBG controller. The Denavit–Hartenberg (DH) model confirms that movement is generated at the corresponding joint, not at the wrist. Additionally, the end-effector is not located at the hand but directly beneath the flexion–extension mechanism, which alters the kinematic chain. The angular velocity (red curve), which is the derivative of *θ*_1_, follows a bell-shaped profile characteristic of TBG control (Figure 9), ensuring smooth transitions. From the DH perspective, this configuration enables a controlled motion without abrupt accelerations, reducing mechanical stress on the structure. The homogeneous transformation matrix derived from the DH parameters confirms that the end-effector follows the intended trajectory, maintaining both position and orientation as expected. Implementing SMC + TBG within this modified DH framework enhances system stability and prevents unnecessary strain on the components. In conclusion, the simulation with SMC + TBG demonstrates efficient control of the two-DOF prostheses, validated by Denavit–Hartenberg modeling, ensuring a natural and biomechanically coherent movement adapted to its unique configuration.

In Figure 9, smooth angular motion with symmetrical acceleration and deceleration is shown, which aligns with SMC + TBG’s biomimetic control strategy. Angular velocity peaks during rapid displacement changes and zeroes at displacement peaks, mimicking the natural movements of human joints. SMC + TBG generates bell-shaped velocity profiles, which ensure stable and precise motion under nonlinear dynamics. This approach enhances natural movement and user comfort in elbow prostheses.

**Figure 10:**
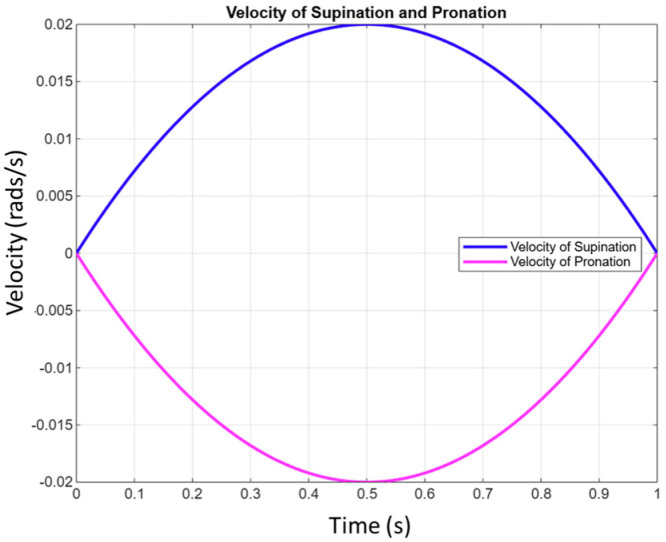
The velocity results of the prosthesis.

**Figure 11:**
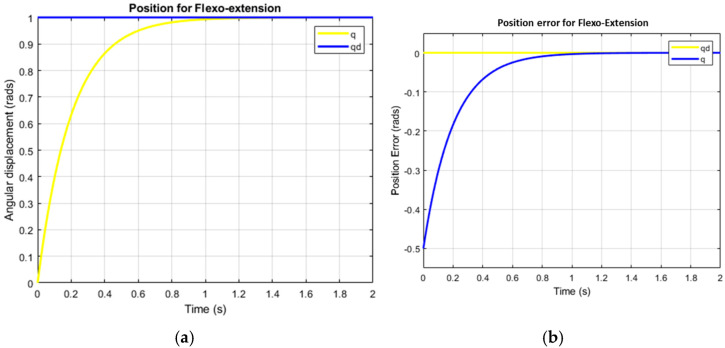
The angular displacement profile and error of the prosthesis under flexion–extension.

**Figure 12:**
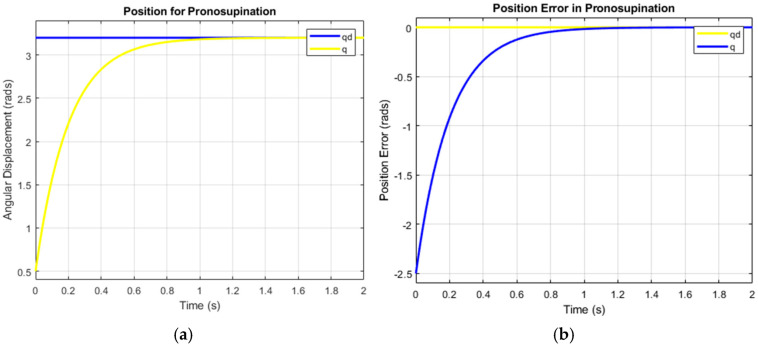
The angular displacement profile and error of the prosthesis under pronation–supination.

Figure 10 illustrates the system’s ability to replicate complex human movements with smooth, continuous control that reflects both actuator behavior and human body limitations. The flexion–extension graph shows bell-shaped, coordinated motion (Figure 9), while the pronation–supination graph, highlighting faster pronation than supination, highlights adaptive control. This modulation enhances the realistic replication of human motion. The flexion–extension movement exhibits high synchronization, closely mimicking natural elbow motion, while the pronation–supination movement demonstrates optimized supination, highlighting adaptive control via the SMC + TBG control strategy. This strategy ensures precise trajectory tracking and smooth velocity profiles, effectively emulating human motion with two degrees of freedom. Based on operating cycle measurements, achieving 8 h of autonomy requires a battery capacity of 11,250 mAh. To evaluate control success, error analysis compares desired and obtained positions. Figure 11b and Figure 12b show the error curve smoothly converging to zero, indicating progressive correction and achievement of the desired position. The system stabilizes in approximately 1.2 s for both flexion–extension and pronation–supination, effectively reaching zero error.

In the SMC + TBG scheme proposed in this study, the simulation gains λ = 5 and K = 10 are key to system control. The gain λ controls the convergence speed to the sliding surface in the sliding mode control (SMC). A value of λ = 5 ensures fast convergence but may cause chattering (unwanted oscillations). The gain K = 10 is associated with the equivalent control of the SMC, which keeps the system on the sliding surface. A high value of K provides robustness against disturbances but can also lead to increased chattering.

A time base generator (TBG) generates a smooth reference trajectory, which allows the SMC to operate with a well-defined reference. This helps reduce chattering and improves control smoothness. Although the TBG does not directly yield gains, its output influences the system’s behavior, enabling adjustments to λ and K to balance speed, robustness, and smooth control (Figure 11 and Figure 12). With λ = 5 and K = 10, the system is both fast and robust, but it may exhibit chattering. To mitigate this effect, the gains can be reduced: decreasing λ slows convergence but reduces chattering, while reducing K improves smoothness but may compromise robustness. Optimizing the TBG’s trajectory also helps smooth the motion without drastically reducing the gains. In applications such as a 2-DOF elbow prosthesis, this scheme enables fast and precise movements with the ability to handle variable loads. However, it is crucial to adjust the gains to avoid unwanted vibrations and ensure user comfort. Experimenting with the values of λ and K, along with a smooth TBG trajectory, is essential to achieve robust, precise, and smooth control, as shown in Figure 11 and Figure 12.

**Figure 13:**
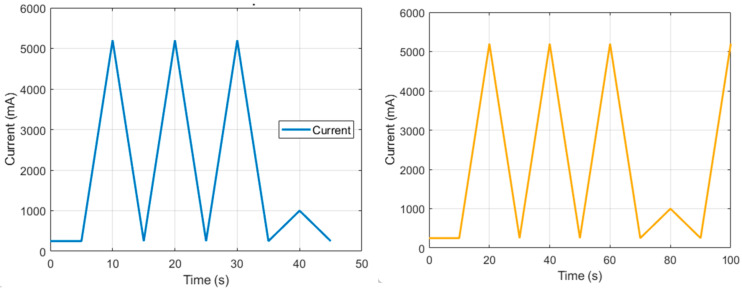
Current results of the prosthesis under pronation (blue)–supination (yellow) motion.

The yellow line shows transient behavior, with the prosthesis position stabilizing at π/3, indicating adequate damping and stable response. Figure 11a and Figure 12a confirm overdamping (above 1 at t = 1.4) as the real positions converge with the desired positions: 60° for flexion–extension and 30° for supination. Rapid stabilization ensures intuitive user perception, while joint behavior reflects system stability and predictability. These results demonstrate precise, stable, and user-friendly motion control. In Figure 13, the high current peaks indicate the moments when the system requires more power. These peaks correspond to the instances where motion is executed, such as when the actuator changes direction or undergoes significant acceleration. The low current intervals between the peaks represent the moments when the actuator is not working intensively, possibly in standby mode, or moving at a very slow pace, which correlates with lower energy consumption.

**Figure 14:**
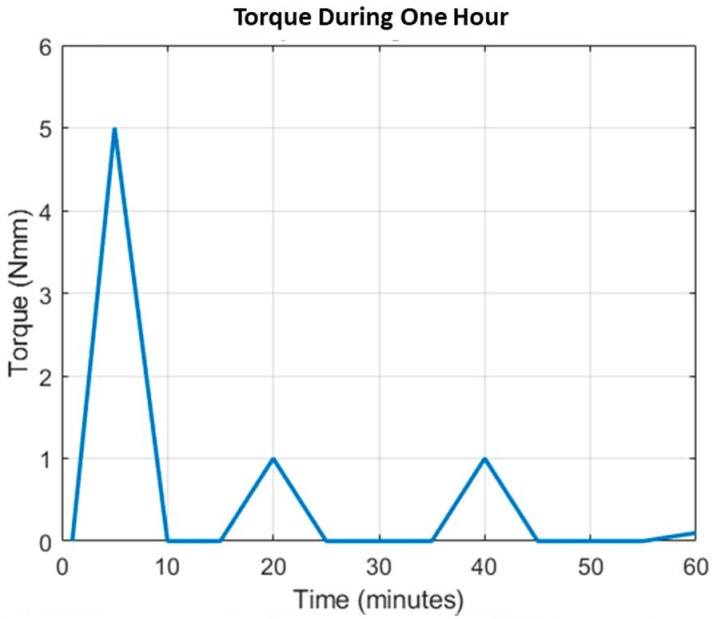
The torque results of the prosthesis over the course of 1 h.

A representative time plot was constructed, in which the displacement and velocity were expressed for the movement repetitions. The graphs in Figure 11 and Figure 12 show the flexo-extension and pronation–supination profiles, which demonstrate that both movements have the same displacement during the performance of the prosthesis. Figure 14 shows torque spikes corresponding to movement cycles of the 2-DOF elbow prosthesis, with the highest peak occurring within the first 5 min. This behavior reflects efficient control using SMC + TBG for smooth trajectory tracking. However, the 450 mAh battery is insufficient, as peak performance is reached within 20 min, and the system requires an 11,250 mAh battery. To meet this demand, four 3000 mAh batteries are recommended, ensuring continuous operation and improved autonomy.

While the PID controller focuses on modulating the current to achieve stability and precision, the SMC-TBG approach introduces a time-based trajectory generation that ensures smoother and more natural movements. The SMC component of SMC + TBG provides robustness against uncertainties and disturbances, ensuring that the system maintains precise control even under varying conditions. Meanwhile, the TBG component generates biomimetic trajectories with bell-shaped velocity profiles, which are crucial for replicating human-like motion patterns. The integration of SMC + TBG with the PID controller optimizes the relationship between the signal and the current, leading to smoother operation and improved energy efficiency.

By reducing abrupt changes in current, SMC + TBG minimizes mechanical wear and enhances the actuator’s performance, making it particularly suitable for applications requiring precise and natural movements, such as prosthetics or robotic systems. The implementation of the SMC + TBG ensures that the actuator can adapt to varying conditions while maintaining optimal performance, providing a superior control strategy for advanced applications. Efficiency and system response are critical, as current graphs reveal latency in Bluetooth signal reception, which impacts movement accuracy. Proper current modulation, synchronized with voltage, ensures precise motion within specified ranges, optimizing performance. This balance is key for accurate and efficient system operation.

**Figure 15:**
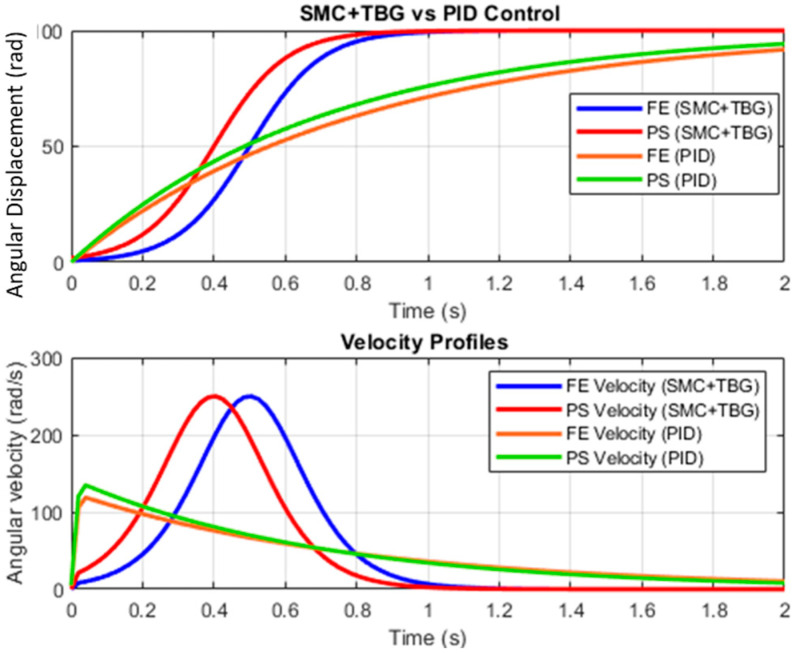
Results of angular displacement versus time and angular velocity versus time under flexion–extension (FE) and pronation–supination (PS), with the control method of the prosthesis being either SMC + TBG or PID.

Figure 15 shows the angular displacement and angular velocity results for each movement. In the case of pronation–supination, there is a change in the direction of rotation. The sliding mode control complements the PID controller with time base generation (SMC + TBG), which enhances the system’s performance by providing a more robust and adaptive control strategy. Figure 15 shows the development of trajectories without the SMC + TBG control method and with it in the prosthesis, using the work developed in this study as a reference. This study controls the elbow joint movement using PID control for rehabilitation and proposes the SMC + TBG. Comparison of PID and SMC + TBG control methods: The control of a 2-DOF elbow prosthesis, which performs flexion–extension (FE) and pronation–supination (PS), is analyzed using two strategies: PID and sliding mode control with time base generator (SMC + TBG). With the SMC + TBG control strategy, the angular displacement and angular velocity profiles achieved for the prosthesis were faster than those obtained using PID control (Figure 15).

SMC + TBG generates sigmoidal trajectories that reduce abrupt accelerations, minimizing mechanical stress and enhancing comfort. The velocity profiles confirm that SMC + TBG maintains a bell-shaped curve, which is crucial for natural motion. In contrast, PID produces a more linear response, which may increase energy consumption and reduce adaptability to external disturbances. The jerk analysis reveals that SMC + TBG significantly reduces sudden variations in acceleration compared to PID, which exhibits higher discontinuities. Lower jerk values improve user comfort and reduce wear on prosthetic components. These findings suggest that SMC + TBG is more efficient in movements. Real-time implementation of this method will be explored in a future study.

## 4. Conclusions

This work focused on the mechanical design, development of a control system, and laboratory evaluation of the performance of a two-degrees-of-freedom elbow prosthesis. Control was achieved using either SMC + TBG or PID. During the evaluation, the current, angular displacement, and angular velocity of the prosthesis during flexion–extension and pronation–supination, each as a function of time, were obtained. With control using SMC + TDG, the obtained angular displacement and angular velocity results for the prosthesis are within the range of a healthy adult. Thus, the results suggest that the prosthesis in which control is achieved using the SMC + TBG strategy shows promise for use by transhumeral amputees.

The development and evaluation of a two-degrees-of-freedom (DOF) elbow prosthesis represent a significant advancement in prosthetic technology, enabling flexion–extension and pronation–supination movements within normal physiological ranges, suggesting an effective biomechanical design and precise motion control. This research highlights the importance of controlled current modulation for actuator function, initially implementing a PID controller that provides a stable basis for flexion–extension movements. However, the integration of sliding mode control with time base generation (SMC + TBG) demonstrates a substantial improvement in the control strategy.

The comparison of the two control methods shows that SMC + TBG has four advantages. First, SMC exhibits greater precision in trajectory tracking and enhanced adaptability to environmental variations and loads, in contrast to the stability offered by the PID controller. Second, the combination of TBG to generate biomimetic trajectories with smooth velocity profiles, along with the precise control of SMC, results in more fluid and natural movements, which are crucial for prosthetic functionality. Third, the optimized current control achieved using SMCTBG contributes to minimizing mechanical wear and improving the energy efficiency of the prosthesis. Fourth, the synergy between TBG and SMC minimizes “jerking”, which results in less abrupt and more comfortable movements.

The present results indicate that using the SMC + TGB control strategy for a two-degrees-of-freedom elbow prosthesis has good potential to ensure natural movements of the prosthesis. The results point the way to future developments, such as further evaluation of this control strategy and, depending on the outcome of that evaluation, its incorporation into the next generation of the two-degrees-of-freedom elbow prostheses for use by transhumeral amputees.

This study focused on the design, control, and laboratory testing of a 2DOF elbow prosthesis using SMC + TBG and PID strategies. Although clinical trials were not conducted, the aim was first to validate the system in controlled environments, making this an acceptable first step. No comparison was made with healthy human motion due to time constraints; however, the prosthesis produced smooth, biomimetic trajectories aligned with those reported in the literature. The battery used (450 mAh) was limited but sufficient for demonstrating feasibility; future versions will address autonomy. PLA and commercial components were selected for rapid prototyping, ensuring functional validation was not compromised. Finally, simplified dynamic models were used to allow real-time control; while not anatomically exhaustive, they effectively supported accurate motion replication. These limitations do not affect the core findings, which confirm the viability of the proposed design and control strategy.

## 5. Patents

As a result of this research study, a patent for the design of a two-degrees-of-freedom elbow prosthesis has been granted. Registrations were obtained in Mexico City (MX/f/2023/003784).

## Figures and Tables

**Table 1:** The model definition for the application of the Denavit–Hartenberg equations.

Link	θi (Joint Angle)	Di (Displacement Along z2)	ai (Length of the Link Along ×2)	α_i_ (Angle Between Zi and Zi + i)
1 (Flexo-extension)	$\theta_{1}$	0	$\alpha_{i}$	$90^{{}^{\circ}}$
2 (Pronosupination)	$\theta_{2}$	0	$\alpha_{ii}$	$0^{{}^{\circ}}$

## Data Availability

Data are contained within the article.

## Abbreviations

**Acronyms**	**Definition**
PID	Proportional–integral–derivative control strategy
DOF	Degrees of freedom
SMC	Sliding mode control
TBG	Time base generator
PWM	Pulse width modulation
PLA	Polylactic acid
**Symbol**	**Definition**
**τ**	Torque
**k**	Stiffness constant
**Λ**	Constant that weights the position vector
**q**	Position
**N**	Gears
**L**	Lagrangian of the system
**θ**	Angle
**α**	Angle
**τC**	Control torque vector applied to the robot’s joints.
**Kp**	Proportional gain matrix.
**Kd**	Proportional derivative gain matrix
**e = q − qr**	Posotion error
**e˙= q˙ − q˙r**	Joint velocity error
**G(q)**	Gravity torque
